# High Prevalence of Viral Infections Among Hospitalized Pneumonia Patients in Equatorial Sarawak, Malaysia

**DOI:** 10.1093/ofid/ofz074

**Published:** 2019-02-13

**Authors:** Teck-Hock Toh, King-Ching Hii, Jane K Fieldhouse, Jakie Ting, Antoinette Berita, Tham Thi Nguyen, See-Chang Wong, Toh-Mee Wong, Wei-Honn Lim, Siaw-Jing Ha, Chuet-Zou Lau, Sing-Ling Kong, Emily S Bailey, Tyler E Warkentien, Tupur S Husain, Gregory C Gray

**Affiliations:** 1 Clinical Research Center, Sibu Hospital, Ministry of Health Malaysia, Sarawak; 2 Department of Paediatrics, Sibu Hospital, Ministry of Health Malaysia, Sarawak; 3 Department of Medicine, Sibu Hospital, Ministry of Health Malaysia, Sarawak; 4 Faculty of Medicine, SEGi University, Kota Damansara, Selangor, Malaysia; 5 Department of Paediatrics, Kapit Hospital, Ministry of Health Malaysia, Sarawak, Malaysia; 6 Duke Global Health Institute, Duke University, Durham, North Carolina; 7 Division of Infectious Diseases, Duke University School of Medicine, Durham, North Carolina; 8 Emerging Infectious Disease Program, Duke-NUS Medical School, Singapore; 9 Naval Medical Research Center-Asia, Singapore

**Keywords:** adenoviruses, influenza, pneumonia, respiratory syncytial virus, respiratory viruses

## Abstract

**Background:**

Although pneumonia is a known cause of morbidity and mortality in Sarawak, Malaysia, the etiology and epidemiology of pneumonia are not well described in this equatorial region. Routine clinical diagnostics for pneumonia etiology at government hospitals in Sarawak had historically involved only bacterial diagnostics. Viral diagnostics were only obtained through outside consultations.

**Methods:**

From June 15, 2017 to May 14, 2018, we collected nasopharyngeal swabs from 600 patients of all ages older than 1 month hospitalized with pneumonia at Sibu and Kapit Hospitals. Specimens were examined at our collaborating institutions with a panel of molecular assays for viral pathogens including influenza A (IAV), IBV, ICV, and IDV, human adenovirus (AdV), human enterovirus (EV), human coronavirus (CoV), respiratory syncytial virus subtype A (RSV-A) or RSV-B, and parainfluenza virus (PIV) types 1–4.

**Results:**

Of 599 samples examined, 288 (48%) had molecular evidence of 1 or more respiratory viruses. Overall, the most prevalent virus detected was RSV-A (14.2%) followed by AdV (10.4%) and IAV (10.4%), then RSV-B (6.2%), EV (4.2%), IBV (2.2%), PIV-3 (1.7%), CoV (1.0%), PIV-1 (1.0%), PIV-4 (0.7%), and PIV-2 (0.2%). No specimens were confirmed positive for ICV or IDV.

**Conclusions:**

The high prevalence of viruses detected in this study suggest that respiratory viruses may be responsible for considerable morbidity in equatorial regions such as Sarawak. Access to viral diagnostics are very necessary for medical staff to determine appropriate pneumonia treatments.

As viral respiratory tract infectious diseases such as avian influenzas and severe acute respiratory syndrome coronavirus have emerged as increasing threats to global health security, international stakeholders such as the World Health Organization and the United Nations International Children's Emergency Fund (UNICEF) are calling for increased global surveillance for emerging and re-emerging respiratory viruses [1–3]. Local-level surveillance systems are critical to understanding and monitoring the epidemiology of severe respiratory disease. Understanding the etiology and epidemiology of pneumonia will guide antiviral interventions as well as enhance global emerging infectious disease preparedness.

Pneumonia is the second leading cause of death among children under the age of 5, resulting in more childhood deaths than diarrhea and malaria combined, with more than 95% of those cases occurring in low- and middle-income countries [4, 5]. A 2015 meta-analysis of the global burden of childhood pneumonia found the greatest proportion (39%) of severe pneumonia cases occurred in Southeast Asia [6]. However, because *Streptoccocus pneumoniae* is the most common cause of vaccine-preventable severe pneumonia, much of the literature has focused on bacterial causes of pneumonia or antibiotic resistance, whereas fewer studies have prospectively investigated the viral etiology of pneumonia in Southeast Asia [7–9]. One study of respiratory samples collected from children living in Kuala Lumpur under 5 years of age between 1982 and 2008 found that 26.4% of the samples were positive by immunofluorescence assays and viral cultures for viral pathogens, with a prevalence of 18.6% for respiratory syncytial virus (RSV), 3.5% for parainfluenza viruses (PIVs), 2.9% for influenza viruses, and 1.37% for adenovirus [10].

During the past 5 years, the directors of 2 government hospitals (Sibu Hospital and Kapit Hospital) in their namesake cities in Sarawak, Malaysia, have seen increasing pneumonia admissions (see Supplementary Figure 1). Sibu Hospital, a referral hospital for central Sarawak serving a population of approximately 725 400 [11], experienced 1611 pneumonia admissions in 2013, 1607 admissions in 2014, and 1903 admissions in 2015. Kapit Hospital serves a smaller population of approximately 130 800 residents living along the Rejang River [11], 120 kilometers east and inland from Sibu. The hospital has similarly experienced a recent increase in pneumonia admissions, estimated at more than 300 per year.

Before this study, routine clinical diagnostics for pneumonia etiology at both Sibu and Kapit Hospitals involved chiefly blood culture and Gram stains of endotracheal secretion, if intubated, to detect bacteria. Molecular and immunofluorescence laboratory diagnostics were only ordered for severe cases, for which clinicians had to send specimens to a specialized virology laboratory in Kuala Lumpur, Malaysia or the University Centre in Kuching, Sarawak. The overall objective of this study was to examine the viral etiology of and risk factors for pneumonia among patients admitted to Sibu and Kapit Hospitals between June 2017 and May 2018 and, in doing so, to assist Malaysian collaborators with setting up sustainable real-time molecular assays for viral respiratory pathogens.

## METHODS

### Participants

Study enrollment took place between June 2017 and May 2018 at Sibu and Kapit Hospitals. The major ethnic groups in Sarawak are Iban, Chinese, Malay, Melanau, Bidayuh, and Orang Ulu. As of 2016, approximately 37.8% of the population in Sibu was Iban and 60.7% of the population in Kapit was Iban [11].

As previously described [12], we adapted inclusion and exclusion criteria from 2 United States, large and comprehensive, community-based pneumonia studies published in 2015 [13, 14]. All patients older than 30 days admitted to Sibu or Kapit Hospitals and diagnosed with pneumonia by an attending physician were considered for study eligibility. A Medical Officer (MO) evaluated eligible subjects for inclusion and exclusion criteria, including confirmation by chest radiography within 72 hours of hospitalization (see Supplementary Table 1).

Adults 18 years of age or older provided written consent, whereas children ages 7 to 18 provided written assent along with written parental or guardian consent. Written consent was obtained from all parents or guardians of children under the age of 7. The study received a scientific review, and all procedures followed were in accordance with the ethical standards of the Malaysian Ministry of Health’s Medical Research and Ethics Committee (protocol number NMRR-17-316-34395), the Duke University Health System Institutional Review Board, Duke-NUS Medical School Ethical Review Board, and the Naval Medical Research Center-Asia Human Research Protection Program (HRPO no. W911QY-16-D-0058).

Although this pilot study had sparse Sarawak baseline virus prevalence data from which to calculate sample size, we approximated sample size based upon an estimated prevalence of influenza A (IAV) causing 30% of pneumonias. Hence, if we enrolled 600 pneumonia patients, we could be confident to calculate an estimated prevalence within 3.8% of the true prevalence of IAV.

### Procedures

From June 15 to July 27, 2017, the study team relied on convenience sampling to enroll as many patients as possible. For the remaining 10 months of the study, MOs enrolled patients on 2 of 3 randomly selected days of the week, which were communicated to them by a study coordinator.

After obtaining written assent and/or consent, MOs administered a brief questionnaire. The questionnaire captured demographic information (age, gender, ethnicity) along with household size (number of cohabitants) and contact with animals within the last 30 days. In addition, all pre-existing health conditions and current medications were self-reported by the subject or their parent/guardian during the questionnaire, then confirmed by the MO through a review of the patient’s medical records.

The MO then used a flocked swab to collect 1 nasopharyngeal (NP) swab from the subject’s nose, which was quickly placed into a viral transport tube containing 3 mL sterile viral transport medium (BD Universal Viral Transport; Becton, Dickinson and Company, Franklin Lakes, NJ).

### Ribonucleic Acid and Deoxyribonucleic Acid Extraction and Real-Time Reverse-Transcription Polymerase Chain Reaction (PCR)/Real-Time PCR

All specimens were stored at −80°C at the Sibu Hospital Clinical Research Center (SHCRC) until ribonucleic acid or deoxyribonucleic acid extraction was performed using the QIAmp Cador Pathogen Mini Kit (QIAGEN, Hilden, Germany). Specimens collected in Kapit were stored at −80°C until they could be transported to Sibu for processing. Real-time reverse-transcription polymerase chain reaction (rRT-PCR) or real-time PCR (rPCR) was conducted on similar BioRad CFx96 C1000 Touch Thermal Cycler Real-Time systems at SHCRC, Duke-NUS in Singapore and at Duke University in Durham, North Carolina.

All NP swabs were examined for molecular evidence of influenza A (IAV), IBV, ICV, and IDV, human adenovirus (AdV), human enterovirus (EV), human coronavirus (CoV), RSV subtype A (RSV-A) and RSV-B, and PIV types 1–4 (PIV-1, -2, -3, and -4). Primer and probes sequences for targeted pathogens are recorded in Supplementary Table 2. A positive control and a no-template control were included in each run. All specimens were first run at SHCRC. One-milliliter aliquots of these specimens were later shipped on dry ice to either Duke-NUS or Duke University for validation. Laboratory staff at Duke-NUS and Duke were blind to the results of the assays at SHCRC. Cycle threshold (Ct) values <38 were considered positive, and Ct values 38 to 40 were considered suspect to acknowledge that positivity could be the result of cross-reactivity or nonspecific amplification. Cycle threshold values >40 were considered negative. All discrepant assays were noted and repeated at both SHCRC and either Duke or Duke-NUS, using the same original assays, and discrepancies were thus resolved. Partial genome sequencing was also performed for several specimens to validate discrepant results.

### Statistical Analyses

Questionnaire data and molecular results were entered into REDCap version 7.0 and verified at SHCRC and at Duke. Data were imported into STATA version 15.0 (StataCorp, College Station, TX), cleaned, and categorized for statistical analyses. We categorized continuous variables based on the distribution of the counts for household size (quartiles) and for age (quartiles). After examining age quartiles, we rounded age categories into approximate age quartiles so that age was treated as a whole number. Demographic and clinical risk factors were examined for bivariate associations with the most prevalent positive molecular assays. Pearson’s χ^2^ test or Fishers exact test were used for bivariate work. Risk factors with a bivariate test statistic *P* ≤ .1 were included in stepwise, manual, backward-elimination, unconditional logistic regression models. Risk factors with *P* < .05 were retained in final models, and odds ratios (ORs) and 95% confidence intervals (CIs) were calculated.

## RESULTS

A total of 600 hospitalized pneumonia patients were enrolled at Sibu and Kapit Hospitals between June 15, 2017 and May 15, 2018, with 389 of the subjects enrolled at Sibu Hospital (64.8%) and 211 enrolled at Kapit Hospital (35.2%). Of the enrolled subjects, 325 (54.2%) were male. A total of 385 (64.2%) enrolled subjects were children 5 years of age or younger and 439 (73.2%) were of age 18 years and younger. The majority of participants identified as Iban, with 253 (65.0%) Iban subjects enrolled at Sibu Hospital and 188 (89.1%) Iban subjects enrolled at Kapit Hospital (Table 1).

**Table 1.** Demographic Characteristics and Exposure Variables Reported Upon Enrollment Among Patients Hospitalized With Pneumonia at Sibu and Kapit Hospitals, Between June 2017 and May 2018

Exposure Variables	All Total No. (%)	Sibu No. (%)	Kapit No. (%)
Total	600 (100)	389 (64.8)	211 (35.2)
Gender			
Female	274 (45.7)	187 (48.1)	87 (41.2)
Male	326 (54.3)	202 (51.9)	124 (58.8)
Age Group (Approximate^a^ Quartiles)			
0–1 years (µ 0.5)	180 (30.0)	93 (23.9)	87 (41.2)
1–2 years (µ 1.4)	105 (17.5)	60 (15.4)	45 (21.3)
2–18 years (µ 6.4)	154 (25.7)	94 (24.2)	60 (28.4)
>18 years (µ 63.9)	161 (26.8)	142 (36.5)	19 (9.0)
Household Size (Quartiles)			
0–3 (µ 2.0)	161 (26.8)	113 (29.0)	48 (22.8)
4–5 (µ 4.5)	178 (29.7)	126 (32.4)	52 (24.6)
6–7 (µ 6.4)	133 (22.2)	71 (18.3)	62 (29.4)
≥8 (µ 11.2)	128 (21.3)	79 (20.3)	49 (23.2)
Ethnicity			
Iban	441 (73.5)	253 (65.0)	188 (89.1)
Malay	42 (7.0)	36 (9.3)	6 (2.8)
Chinese	46 (7.7)	43 (11.1)	3 (1.4)
Other	71 (11.8)	57 (14.7)	14 (06.6)
Pre-existing Conditions			
Hypertension	89 (14.8)	76 (19.5)	13 (6.2)
Diabetes mellitus	37 (6.2)	31 (8.0)	6 (2.8)
Lung disease	116 (19.3)	112 (28.8)	4 (1.9)
Other	60 (10.0)	54 (13.9)	6 (2.8)
None	389 (64.8)	197 (50.6)	192 (91.0)
Medical Treatment in Last 6 Months			
Diabetes medicine	36 (6.0)	30 (6.5)	6 (3.4)
Hypertension medicine	84 (14.0)	73 (14.3)	11 (12.5)
Corticosteroids	93 (15.5)	86 (22.1)	7 (3.3)
Other	48 (8.0)	47 (12.1)	1 (0.5)
None	405 (67.5)	211 (54.2)	194 (91.9)
Animal Contact^b^			
Pigs	18 (3.0)	15 (3.9)	3 (1.4)
Chickens	68 (11.3)	41 (10.5)	27 (12.8)
Ducks and other poultry	33 (5.5)	11 (2.8)	2 (1.0)
** **Cats	141 (23.7)	117 (30.0)	24 (11.4)
Dogs	73 (12.2)	57 (14.7)	16 (7.6)
Other^c^	177 (29.5)	144 (37.0)	33 (15.6)
None	326 (54.3)	117 (30.1)	149 (70.6)
Month of Enrollment			
June 15–July 14	97 (16.2)	53 (13.6)	44 (20.9)
July 15–August 14	51 (8.5)	43 (11.1)	8 (3.8)
August 15–September 14	41 (6.8)	30 (7.7)	11 (5.2)
September 15–October 14	53 (8.9)	28 (7.2)	25 (11.8)
October 15–November 14	47 (7.8)	32 (8.2)	15 (7.1)
November 15–December 14	42 (7.0)	26 (6.7)	16 (7.6)
December 15–January 14	43 (7.2)	27 (6.9)	16 (7.6)
January 15–February 14	71 (11.9)	44 (11.3)	27 (12.8)
February 15–March 14	40 (6.7)	24 (6.2)	16 (7.6)
March 15–April 14	43 (7.2)	28 (7.2)	15 (7.1)
April 15–May 14	72 (12.0)	54 (13.9)	18 (8.5)

Abbreviations: µ, mean age within category.

^a^Rounded to whole years based on age quartiles: 0.06–0.85 (Q1); 0.86–2.16 (Q2); 2.17–25.2 (Q3); 25.3–92 (Q4).

^b^Animal contact defined as touched or come within 1 meter in the last 30 days.

^c^Other animals include the following: cow, rats, rabbits, snake, and monkey.

**Figure 1.**
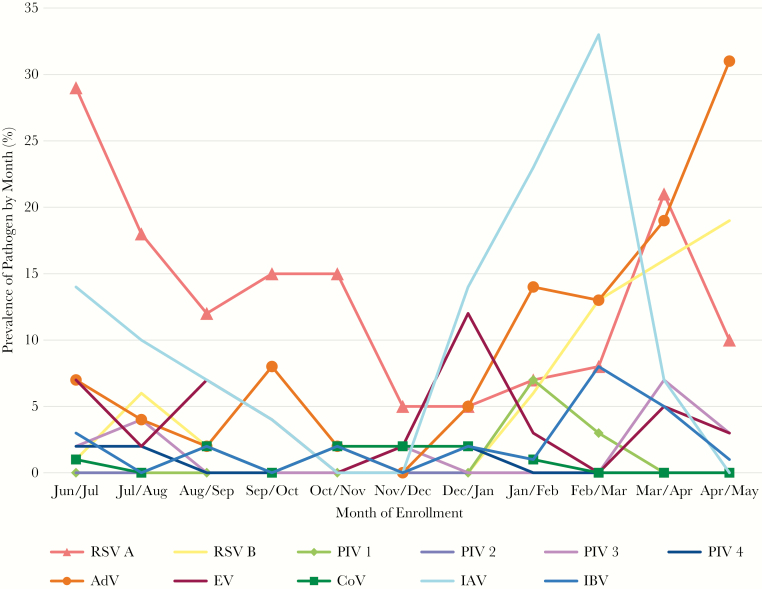
Prevalence of pathogens detected by month, patients hospitalized with pneumonia in Sibu and Kapit hospitals, June 15, 2017–May 14, 2018. AdV, human adenovirus; CoV, human coronavirus; EV, human enterovirus; IAV, influenza A; IBV, influenza B; PIV, parainfluenza virus; RSV, respiratory syncytial virus.

Of the 600 NP swabs collected, 599 were run using rRT-PCR/rPCR; 1 NP swab collected from a pediatric patient at Sibu Hospital was accidentally destroyed before molecular screening. One or more viruses were detected by rRT-PCR/rPCR in 48.1% of the 599 samples testing positive or suspect positive for either IAV, IBV, ICV, IDV, AdV, EV, CoV, RSV-A, RSV-B, PIV-1, PIV-2, PIV-3, or PIV-4. The prevalence of each pathogen out of the total number of patients enrolled by month is shown in Figure 1.

 We detected coinfections in 42 of the 275 patients with evidence of viral infection (Table 2). Of the coinfected specimens, AdV was detected in approximately 65% (n = 27) with the most common coinfection combination being AdV and RSV (9 AdV/RSV-B coinfections and 6 AdV/RSV-A coinfections) followed by 7 AdV/EV coinfections. Respiratory syncytial virus subtype B was the second most prevalent virus among coinfections (n = 15); in addition to coinfections with AdV-positive specimens, RSV-B was also detected in specimens found positive by rRT-PCR for RSV-A, PIV-1, EV, IAV, and IBV. One pediatric patient’s specimen was positive for 3 viruses (RSV-B, AdV, and EV).

**Table 2.** Single Virus Infection Versus Coinfection Prevalence Detected by rRT-PCR or rPCR^a^

Virus	No. Total Positive (%Total Specimens^b^)	Single Virus Infection (%Total Positive)	Coinfection n (%Total Positive)
Respiratory syncytial virus A	85 (14.2)	71 (83.5)	14 (16.5)
Adenovirus	62 (10.4)	35 (56.5)	27 (43.5)
Influenza A virus	62 (10.4)	53 (85.5)	9 (14.5)
Respiratory syncytial virus B	37 (6.0)	22 (59.5)	15 (40.5)
Enterovirus	25 (3.3)	15 (60.0)	10 (40.0)
Influenza B virus	13 (2.2)	10 (76.9)	3 (23.1)
Parainfluenza 3	10 (1.7)	9 (90.0)	1 (10.0)
Coronavirus	6 (1.0)	5 (83.3)	1 (16.7)
Parainfluenza 1	6 (1.0)	3 (50.0)	3 (50.0)
Parainfluenza 4	4 (0.7)	4 (100.0)	0 (0.0)
Parainfluenza 2	1 (0.2)	1 (100.0)	0 (0.0)
Influenza C virus	0 (0.0)	0 (0.0)	0 (0.0)
Influenza D virus	0 (0.0)	0 (0.0)	0 (0.0)

Abbreviations: rPCR, real-time reverse polymerase chain reaction; rRT-PCR, real-time reverse-transcription PCR.

^a^Does not include suspect positives.

^b^Denominator is total number of specimens examined by rRT-PCR and rPCR for viruses, n = 599.

Overall, the most prevalent virus detected was RSV (Table 3), with 122 samples testing positive for either RSV-A or RSV-B (overall prevalence of 20.4%; prevalence of 26.9% among children <18 years). A total of 85 specimens tested positive by rRT-PCR for RSV-A for an overall prevalence of 14.2%. An additional 7 specimens were suspect-positive for RSV-A (Ct values ranged 38.3–39.7); however, suspect-positive specimens were not included in the final analyses. Eighty-two of the RSV-A-positive samples were from children ≤5 years age 5, totaling a prevalence of 21.4%. A total of 37 (6.2% prevalence) specimens were positive for RSV-B by rRT-PCR, with 2 additional suspect-positive specimens (Ct values ranged 38.9–39.6). Thirty-five of those specimens were collected from children <18 years (8.0% prevalence).

**Table 3.** Molecular Assay Results Among Children and Adults for All Viruses by rRT-PCR or rPCR Overall and by Children <18 Years of Age and Adults >18 Years of Age^a^

Virus	Overall	Children <18 Years			Adults ≥18 Years		
	Total n = 599 (%)	Total n = 438 (%)	Sibu n = 246 (%)	Kapit n = 192 (%)	Total n = 161 (%)	Sibu n = 142 (%)	Kapit n = 19 (%)
Respiratory syncytial virus A	85 (14.2)	83 (19.0)	43 (17.5)	40 (20.8)	2 (01.2)	1 (00.7)	1 (05.3)
Adenovirus	62 (10.4)	57 (13.0)	43 (17.5)	14 (7.3)	5 (3.1)	4 (2.8)	1 (5.3)
Influenza A virus	62 (10.4)	46 (10.5)	31 (12.6)	15 (7.8)	16 (9.9)	13 (9.2)	3 (15.8)
Respiratory syncytial virus B	37 (6.2)	35 (8.0)	22 (8.9)	13 (6.8)	2 (1.2)	2 (1.4)	0 (0.0)
Enterovirus	25 (4.2)	23 (5.3)	9 (3.7)	14 (7.3)	2 (1.2)	2 (1.4)	0 (0.0)
Influenza B virus	13 (2.2)	9 (2.1)	8 (3.3)	1 (0.5)	4 (2.5)	3 (2.1)	1 (5.3)
Parainfluenza 3	10 (1.7)	10 (2.3)	8 (3.3)	2 (1.0)	0 (0.0)	0 (0.0)	0 (0.0)
Coronavirus	6 (1.0)	4 (0.9)	1 (0.4)	3 (1.6)	2 (1.2)	2 (1.4)	0 (0.0)
Parainfluenza 1	6 (1.0)	5 (1.1)	2 (0.8)	3 (1.6)	1 (0.6)	0 (0.0)	1 (5.3)
Parainfluenza 4	4 (0.7)	2 (0.5)	1 (0.4)	1 (0.0)	2 (1.2)	2 (1.4)	0 (0.0)
Parainfluenza 2	1 (0.2)	1 (0.2)	0 (0.0)	1 (0.5)	0 (0.0)	0 (0.0)	0 (0.0)
Influenza C virus	0 (0.0)	0 (0.0)	0 (0.0)	0 (0.0)	0 (0.0)	0 (0.0)	0 (0.0)
Influenza D virus	0 (0.0)	0 (0.0)	0 (0.0)	0 (0.0)	0 (0.0)	0 (0.0)	0 (0.0)

Abbreviations: rPCR, real-time reverse polymerase chain reaction; rRT-PCR, real-time reverse-transcription PCR.

^a^Suspect-positive results not included.

After RSV, AdV and IAV were the most prevalent, each with 62 positive specimens detected by rPCR and rRT-PCR, respectively, for an overall prevalence of 10.4%. Fifty-seven of the AdV specimens were collected from children <18 years (13% prevalence), and 46 of the IAV specimens were collected from children <18 years (10.5% prevalence). A lower prevalence (2.2%) of IBV was detected, with 13 IBV-positive specimens, 9 of which were collected from children <18 years of age. Twenty-five specimens were positive for EV with a 4.2% prevalence overall and 5.3% prevalence among children <18 years. Six specimens were positive by rRT-PCR for CoV. Among the PIV types, PIV-3 was the most prevalent, with 10 specimens testing positive for PIV-3, followed by 6 PIV-1 detections, 4 PIV-4 detections, and 1 PIV-2 detection. No specimens were determined to be positive for ICV or IDV; however, there were 2 suspect-positive IDV samples (Ct values ranged 38.2–39.3), which we were unsuccessful at isolating at Duke University.

One patient of the 600 enrolled was pregnant and tested positive for IAV. Three severe adverse events were reported to the Malaysian ethics board for participants who died after enrollment. All 3 deaths were pediatric patients under 1 year of age with negative blood cultures. Human adenovirus was detected in the NP swab specimens of 2 of those patients, 1 of whom had an endotracheal tube culture that grew *Enterobacter aerogenes* before death. Respiratory syncytial virus subtype A was detected in the third patient’s NP swab. No coinfections were detected among these patients.

Due to low counts, CoV, ICV, IDV, and PIV1-4 disease outcomes were not included in the risk factor analysis. Across the 6 disease outcomes examined (RSV-A, RSV-B, IAV, IBV, EV, and AdV), gender was not found as a statistically significant risk factor in the initial bivariate screenings. Additional potential risk factors such as pre-existing diseases, treatment history, and ethnicity were eliminated in the stepwise, backward-elimination multivariate modeling.

The month of enrollment was a statistically significant risk factor for most virus outcomes, including RSV-A, RSV-B, IAV, and AdV (Tables 4 and 5 and Supplementary Tables 3 and 4). Respiratory syncytial virus subtype A was most prevalent during the first month of enrollment, June–July 2017 with an adjusted OR of 7.2 (95% CI, 1.6–33.5) compared with RSV-A detection by rRT-PCR in November–December 2017 (Table 4). In contrast, there was a very low detection of RSV-B in June–July 2017 (see Supplementary Figure 2). Compared to June–July 2017, RSV-B had an increased adjusted OR beginning mid-February, with an adjusted OR of 23.1 (95% CI, 2.9–185.6) between April 15 and May 14, 2018 (Table 5). We detected AdV in over 30% of the 72 specimens collected from April 15 to May 14, 2018. Compared to the month of October–November 2017, there was an increased adjusted OR of 16.9 (95% CI, 2.1–133.4) of AdV infection during the month of April–May.

**Table 4.** Risk Factors for Molecular Detection of Respiratory Syncytial Virus A

Risk Factor	Total N	RSV-A^+^ (%)	Unadjusted OR (95% CI)	Adjusted OR^a^ (95% CI)
Approximate Age Quartiles				
** **1 month–1 year	179^b^	40 (22.3)	22.9 (5.4–96.4)	27.7 (6.4–120.1)
1–2 years	105	28 (26.7)	28.9 (6.7–124.5)	33.8 (7.6–150.8)
2–18 years	154	15 (9.7)	8.6 (1.9–38.2)	9.6 (2.1–43.8)
>18 years	161	2 (1.2)	Ref.	Ref.
Household Size Quartiles				
0–3	161	22 (13.7)	1.2 (0.6–2.3)	2.0 (0.9–4.3)
4–5	177^b^	19 (10.7)	0.9 (0.4–1.8)	1.1 (0.5–2.3)
≥8	128	28 (21.9)	2.0 (1.0–4.0)	2.4 (1.1–5.0)
6–7	133	16 (12.0)	Ref.	Ref.
Month				
June 15–July 14	97	28 (28.9)	8.1 (1.83–35.9)	7.2 (1.6–33.5)
July 15–August 14	51	9 (17.6)	4.3 (0.9–21.1)	4.9 (0.9–25.6)
August 15–September 14	41	5 (12.2)	2.8 (0.5–15.2)	3.6 (0.6–21.2)
September 15–October 14	53	8 (15.1)	3.6 (0.7–17.7)	3.2 (0.6–16.9)
October 15–November 14	46^b^	7 (15.2)	3.6 (0.7–18.4)	4.8 (0.9–26.8)
December 15–January 14	43	2 (4.7)	1.0 (0.1–7.3)	1.1 (0.1–8.7)
January 15–February 14	71	5 (7.0)	1.5 (0.3–8.2)	1.5 (0.3–8.5)
February 15–March 14	40	3 (7.5)	1.6 (0.3–10.3)	1.3 (0.2–8.4)
March 15–April 14	43	9 (20.9)	5.3 (1.1–26.2)	4.0 (0.8–20.7)
April 15–May 14	72	7 (9.7)	2.2 (0.4–10.9)	1.7 (0.3–9.2)
November 15–December 14	42	2 (4.8)	Ref.	Ref.

Abbreviations: CI, confidence interval; OR, odds ratio; Ref., reference; RSV-A, respiratory syncytial virus A.

^a^Adjusted for age quartiles, household size quartile, month of enrollment.

^b^One pediatric patient specimen destroyed, assay results out of n = 599.

**Table 5.** Risk Factors for Molecular Detection of Respiratory Syncytial Virus B

Risk Factor	Total N	RSV-B^+^ (%)	Unadjusted OR (95% CI)	Adjusted OR^a^ (95% CI)
Approximate Age Quartiles				
1 month–1 year	179^a^	20 (11.2)	10 (2.3–43.5)	9.6 (2.1–43.8)
>1–2 years	105	11 (10.5)	9.3 (2.0–42.9)	10.4 (2.1–51.2)
>2–18 years	154	4 (2.6)	2.1 (0.4–11.7)	2.3 (0.4–10.9)
>18 years	161	2 (1.2)	Ref.	Ref.
Household Size Quartiles				
0–3	161	10 (6.2)	2.9 (0.8–10.7)	4.2 (1.1–16.7)
4–5	177^b^	16 (9.0)	4.3 (1.2–15.1)	4.1 (1.1–15.2)
≥8	128	1 (0.8)	2.9 (0.7–11.1)	2.7 (0.7–10.9)
6–7	133	1 (0.8)	Ref.	Ref.
Month				
July 15–August 14	51	3 (5.9)	6.0 (0.6–59.2)	7.9 (0.8–80.2)
August 15–September 14	41	1 (2.4)	2.4 (0.1–39.3)	3.8 (0.2–65.2)
September 15–October 14	53	0 (0.0)	—	—
October 15–November 14	46^b^	1 (2.2)	2.1 (0.1–34.9)	3.0 (0.2–51.8)
November 15–December 14	42	1 (2.4)	2.3 (0.1–38.3)	2.8 (0.2–47.6)
December 15–January 14	43	0 (0.0)	—	—
January 15–February 14	71	4 (5.6)	5.7 (0.6–52.4)	6.5 (0.7–60.6)
February 15–March 14	40	5 (12.5)	13.7 (1.5–121.5)	13.3 (1.4–122.7)
March 15–April 14	43	7 (16.3)	18.7 (2.2–157.1)	19.9 (2.3–172.5)
April 15–May 14	72	14 (19.4)	23.2 (3.0–180.9)	23.1 (2.9–185.6)
June 15–July 14	97	1 (1.0)	Ref.	Ref.

Abbreviations: CI, confidence interval; OR, odds ratio; Ref., reference; RSV-B, respiratory syncytial virus B.

^a^Adjusted for age quartiles, household size quartile, month of enrollment.

^b^One pediatric patient specimen destroyed, assay results out of n = 599.

The only disease outcome for which location of hospitalization (Kapit versus Sibu) was statistically significant was EV; patients with specimens found positive for EV had a 2.4 higher OR (95% CI, 1.1–5.5) of being enrolled at Kapit Hospital compared with Sibu Hospital (see Supplementary Table 5).

Age was a statistically significant risk factor for RSV-A, RSV-B, and AdV, with pediatric patients ages 1 month to 1 year and 1 to 2 years having increased adjusted OR compared with patients >18 years of age. Detection of RSV-A and RSV-B was associated with quartiles of household size (number of additional cohabitants) (Tables 4 and 5).

The only animal exposure that was statistically associated with a disease outcome was cat exposure for IBV, with those patients reporting coming into contact with a cat within 1 meter in the last 30 days having an OR 5.4 times higher (95% CI, 1.8–16.9) than those patients who did not have contact with a cat.

## DISCUSSION

Sarawak, located on the northwest side of the island of Borneo, has an equatorial climate with a high relative humidity that rarely drops below 80% and temperatures ranging from 23°C to 32°C (see Supplementary Figure 2). Both Sibu (2.2873°N, 111.8305°E) and Kapit (1.9951°N, 112.9331°E) are approximately 2 degrees north of the equator (Supplementary Figure 1). The rainy season in this region typically falls between November and February, with December to March seeing the most rain. A drier season typically falls between May and September, with June to August seeing the least rain. Our findings show the peak IAV season falling during that period of the heaviest rains between January and March, with an adjusted odds ratio of 12.3 (95% CI, 2.6–58.4) of positive IAV detection between February 15 and March 14, 2018, compared with IAV detection in September 15–October 15. Previous studies have investigated the relationship between relative humidity or absolute humidity and influenza virus transmission, suggesting that increased levels of humidity are linked with decreased transmission efficiency; however, the prevalence of IAV in Sibu and Kapit Hospitals suggest the high humidity associated with the equatorial climate in this region may not constrain IAV transmission [15, 16].

Our findings regarding the prevalence of RSV support the existing literature that the 2 antigenic subgroups A and B can cocirculate but typically differ by season [17]. Unlike IAV, we saw seasonal variation of RSV-A and RSV-B during the drier months (see Supplementary Figure 3).

The year-long data found a lower prevalence of RSV overall than an earlier analysis of the data collected during the first 2 months of the study, June and July 2017; the logistic regression model from the pilot study also found that hospital location was a risk factor for RSV-A infection, with patients at Kapit Hospital having a higher adjusted OR (adjusted OR = 3.2; 95% CI, 1.3–7.8) of testing positive for RSV-A compared with patients hospitalized at Sibu Hospital [12].

The finding that age was a statistically significant risk factor for RSV infection is consistent with the epidemiology of this virus. In the year-long study, we found a slightly elevated odds ratio of RSV-A and RSV-B infection among children ages 1 to 2 years (RSV-A adjusted OR = 33.8 [95% CI, 7.6–150.8] and RSV-B adjusted OR = 10.4 [95% CI, 2.1–51.2]) compared with children ages 1 month to 1 year (RSV-A adjusted OR = 27.7 [95% CI, 6.4–120.1] and RSV-B adjusted OR = 9.6 [95% CI, 2.1–43.8]).

After the initial statistical analysis, pediatric data were stratified and analyzed for the outcome of RSV-A (see Supplementary Table 6). In the stratified analysis, the same risk factors, including age quartile, household size, and month of enrollment, were found to be risk factors for RSV-A infection. We speculate that the high OR (5.4; 95% CI, 1.8–16.9) observed for the association between cat exposure and IBV (see Supplementary Table 7) may have occurred by chance.

When considering the number of cohabitants in the household of a subject testing positive for RSV-A, Q4 (≥8) had a slightly increased OR compared with Q3 (6 to 7 cohabitants). The same was true when the analysis was run on the stratified data for pediatric subgroups (see Supplementary Table 5). In contrast, for RSV-B infection, Q1 (0 to 3 cohabitants) and Q2 (4 to 5 cohabitants) had slightly increased OR compared with Q3 (4.2 [95% CI, 1.1–16.7] and 4.1 [95% CI, 1.1–15.2], respectively). The mean household size among all enrolled subjects was 5.7 people with a range of 0 to 30 persons. Household size was self-reported and did not capture the type of household (ie, single-family home or traditional longhouse).

The study had a number of limitations. Although the study offered the questionnaire in 3 different languages to ensure comprehension, it is possible that questions about behavior were not accurately understood. After the first 2 months of study, patients were supposedly recruited on 2 randomly selected days of the week; however, we cannot eliminate the possibility that MOs selected the sicker patients in the ward for this study. In addition, this study was limited to hospitalized patients and therefore excluded milder cases of pneumonia not requiring hospitalization. Although we used rigorously validated real-time PCR assays as the gold standard for virus detection, the study was limited to a panel of 13 viruses and we therefore likely missed other important pathogens present in specimens, including human metapneumovirus, rhinovirus, and bacteria. Specifically, this study would benefit from an understanding of the prevalence of bacterial mono- or coinfections within the patient population. This study provides data on 1 year of pneumonia admissions at 2 district government hospitals in Sarawak, Malaysia; however, prevalence of the viruses investigated can vary between years.

Nevertheless, this study is one of the largest viral pneumonia etiology studies of its kind conducted in Southeast Asia. The findings led investigators to extend the study for an additional year, and a subset of 400 samples will be analyzed at Duke University for rhinoviruses A, B, and C. Through this study we found a viral etiology for a relatively high proportion (48.1%) of pneumonia cases, demonstrating the need for these hospitals to gain clinical diagnostics for viral pathogens. This will help hospital medical staff appropriately administer antiviral treatment for illnesses, such as IAV and IBV, and avoid inappropriate use of antibiotics for pneumonia with a viral etiology.

The findings from this study may assist the hospitals in their preparedness for future pneumonia admissions when new antivirals and vaccines are more readily available. Whereas current therapies for RSV infection are largely supportive, as of 2018 there are more than 50 ongoing RSV vaccine trials and 45 RSV antiviral drug trials currently being evaluated, suggesting that new prevention and treatment mechanisms may be on the horizon [2]. The risk factor analysis of age categories and RSV-A detection also provides evidence to support and encourage breastfeeding among newborns.

## CONCLUSIONS

In a 2018 commentary on pneumonia published in *The Lancet*, Watkins and Sridhar [18] describe the multifold factors limiting global action on pneumonia, including the challenges that arise from the complexity of pneumonia due to its multiple etiologies and consequentially diverse treatments. Accurate diagnosis of pneumonia will not only allow clinicians to prescribe better treatment for patients, but it will also contribute to knowledge regarding the respiratory viruses most likely to cause illness in various age groups and communities. Given the high prevalence of viruses detected among the patients enrolled in this study, routine surveillance and more sensitive molecular-based assays for respiratory viruses are recommended for hospitals in Sarawak, Malaysia and similar equatorial climates.

## Supplementary Data

Supplementary materials are available at *Open Forum Infectious Diseases* online. Consisting of data provided by the authors to benefit the reader, the posted materials are not copyedited and are the sole responsibility of the authors, so questions or comments should be addressed to the corresponding author.

ofz074_suppl_supplementary_information

ofz074_suppl_supplementary_figure_1

ofz074_suppl_supplementary_figure_2

ofz074_suppl_supplementary_figure_3

ofz074_suppl_supplementary_table_1

ofz074_suppl_supplementary_table_2

ofz074_suppl_supplementary_table_3

ofz074_suppl_supplementary_table_4

ofz074_suppl_supplementary_table_5

ofz074_suppl_supplementary_table_6

ofz074_suppl_supplementary_table_7
