# Supplementary material for: High Prevalence of Viral Infections Among Hospitalized Pneumonia Patients in Equatorial Sarawak, Malaysia
Source: Open Forum Infect Dis. 2019 Feb 13;6(3):ofz074. doi: 10.1093/ofid/ofz074 (PMC6440682; doi:10.1093/ofid/ofz074)
Supplement: ofz074_suppl_supplementary_information [file ofz074_suppl_supplementary_information.docx]

December 7, 2018

**Supplemental Appendix**

High Prevalence of Viral Infections Among Hospitalized Pneumonia Patients in Equatorial Sarawak, Malaysia

Teck-Hock Toh^1,2,3^, King-Ching Hii^4^, Jane K. Fieldhouse^5,6^, Jakie Ting^1,2^, Antoinette Berita^4^, Tham Thi Nguyen^7^, See-Chang Wong^2,3^, Toh-Mee Wong^1,2,8^, Wei-Honn Lim^1^, Siaw-Jing Ha^2,3^, Chuet-Zou Lau^3^, Sing-Ling Kong^4^, Emily S. Bailey^5,6^, Tyler E. Warkentien^9^, Tupur S. Husain^9^,
and Gregory C. Gray^5,6,7^

^1^ Clinical Research Center, Sibu Hospital, Ministry of Health Malaysia, Sarawak, Malaysia

^2^ Faculty of Medicine, SEGi University, Kota Damansara, Selangor, Malaysia

^3^ Department of Paediatrics, Sibu Hospital, Ministry of Health Malaysia, Sarawak, Malaysia

^4^ Department of Paediatrics, Kapit Hospital, Ministry of Health Malaysia, Kapit, Sarawak, Malaysia

^5^ Duke Global Health Institute, Duke University, Durham, North Carolina, USA

^6^ Division of Infectious Diseases, Duke University School of Medicine, Durham, North Carolina, USA

^7^ Emerging Infectious Disease Program, Duke-NUS Medical School, Singapore

^8^ Department of Medicine, Sibu Hospital, Ministry of Health Malaysia, Sarawak, Malaysia

^9^ Naval Medical Research Center-Asia, Singapore

**Table of contents**

**METHODS page 2**

**TABLES & FIGURES page 3-13**

**REFERENCES page 14**

**Methods**

*Procedures*

## All study instruments were made available in English, Malay, and Mandarin. When administering the brief questionnaire, Medical officers (MOs) would further explain, read or translate questions when necessary. Five (5) MOs from Sibu Hospital and six MOs from Kapit Hospital were trained in the procedures for consenting, enrolling and collecting specimens from subjects. MOs were observed during their first several enrollments to ensure standard operating procedures were followed.

## RNA and DNA Extraction and qRT-PCR/qPCR

The consent form, questionnaire and viral transport medium tube containing the nasopharyngeal swab were labeled with the same study number before the specimen was stored in the ward refrigerator (1-3°C). A study team member collected specimens from the refrigerator and moved them to a -80°C freezer within 24 hours.

Extractions were conducted in a BSL-2 biosafety cabinet using a 10% Distel disinfectant solution as an approved tuberculocidal. Extractions (100μl) were then stored in cryogenic vials in a -80^o^C freezer until ready for further analysis.

All assays were adapted from the literature at Duke University College of Medicine (Duke) in the USA, and later transferred and validated at the Sibu Hospital Clinical Research Centre and Duke-NUS Medical School (Duke-NUS) in Singapore.

**TABLES**

Supplementary Table 1: Inclusion and exclusion criteria checklist used by medical officers while assessing patients for enrollment eligibility

| Inclusion Criteria Children (1 month to 18 years) | Inclusion Criteria Adults (18 years or more) |
| --- | --- |
| - They were admitted to Sibu or Kapit hospital; - Have evidence of acute infection, defined as reported fever or chills, documented fever or hypothermia, or leukocytosis or leukopenia; - Have evidence of an acute respiratory illness, defined as new cough or sputum production, chest pain, dyspnea, tachypnea, abnormal lung examination, or respiratory failure; - A parent or legal guardian provides written informed consent. In addition to parental consent, signed assent document will be sought from children 7 to 18 years of age. - The evidence of illness is consistent with pneumonia as assessed by means of chest radiography within 72 hours before or after admission. | - They were admitted to Sibu or Kapit hospital on the basis of a clinical assessment by the treating clinician; - Have evidence of acute infection, defined as reported fever or chills, documented fever or hypothermia, leukocytosis or leukopenia, or new altered mental status; - Have evidence of an acute respiratory illness, defined as new cough or sputum production, chest pain, dyspnea, tachypnea, abnormal lung examination, or respiratory failure; - Have evidence consistent with pneumonia as assessed by means of chest radiography by the clinical team within 48 hours before or after admission. |
| Exclusion Criteria Children (1 month to 18 years) | Exclusion Criteria Adults (18 years or more) |
| - If they had been hospitalized recently (<7 days for immunocompetent children and <90 days for immunosuppressed children) - If they had already been enrolled in this study within the previous 28 days - If they resided in an extended-care facility - If they had an alternative diagnosis of a respiratory disorder - If they are newborns who never left the hospital - If they have a tracheostomy tube - If they have cystic fibrosis or - If they have cancer with neutropenia - If they have received a solid-organ or hematopoietic stem-cell transplant within the previous 90 days - If they have active graft-versus-host disease or bronchiolitis obliterans - If they have human immunodeficiency virus infection with a CD4 cell count of less than 200 per cubic millimeter (or a percentage of CD4 cells <14%). | - If they had been hospitalized recently (<28 days for immunocompetent patients and <90 days for immunosuppressed patients), - If they have already been enrolled in this study within the previous 28 days - If they were functionally dependent nursing home residents, - If they have a clear alternative diagnosis - If they have undergone tracheotomy - If they have a percutaneous endoscopic gastrostomy tube - If they have cystic fibrosis - If they have cancer with neutropenia, - If they have received a solid-organ or hematopoietic stem-cell transplant within the previous 90 days, - If they have active graft-versus-host disease - If they have bronchiolitis obliterans - If they have human immunodeficiency virus infection with a CD4 cell count of less than 200 per cubic millimeter. |

Adapted from: Jain S et al. 2015;372(9) [1] and Jain S et al. 2015;373(5) [2].

Supplementary Table 2: Primers and probes sequences and gene target region for all rRT-PCR and rPRC assays. All assays were adapted from the cited references at Duke University.

| ***Virus Assay*** | ***Function*** | ***Sequence*** | ***Target Gene*** |
| --- | --- | --- | --- |
| Influenza A [3] | Forward Primer | 5-GAC-CRA-TCC-TGT-CAC-CTC-TGA-C-3 | Matrix |
|  | Reverse Primer | 5-AGG-GCA-TTY-TGG-ACA-AAK-CGT-CTA-3 |  |
|  | Probe | 5-FAM-TGC-AGT-CCT-CGC-TCA-CTG-GGC-ACG-BHQ 1-3 |  |
| Influenza B [3] | Forward Primer | 5-TCC-TCA-AYT-CAC-TCT-TCG-AGC-G-3 | Matrix |
|  | Reverse Primer | 5-CGG-TGC-TCT-TGA-CCA-AAT-TGG-3 |  |
|  | Probe | 5-FAM-CCA-ATT-CGA-GCA-GCT-GAA-ACT-GCG-GTG-BHQ 1-3 |  |
| Influenza C [4] | Forward Primer | 5’-TGGGAGAGATGGTGTGGAGATA-3’ | Matrix |
|  | Reverse Primer | 5’-TCTTTTTCCATCGAGTCAATTTCA-3’ |  |
|  | Probe | 5’-FAM-AAAGACCACAATTATGC-IBFQ-3’ |  |
| Influenza D [5] | Forward Primer | 5’-GCTGTTTGCAAGTTGATGGG-3’ | PB1 |
|  | Reverse Primer | 5’-TGAAAGCAGGTAACTCCAAGG-3’ |  |
|  | Probe | 5’-FAM-TTCAGGCAAGCACCCGTAGGATT-IBFQ-3’ |  |
| Human Adenovirus [6] | Forward | 5-CAG-GAC-GCY-TCG-GAG-TAC-CTG-A-3 | Hexon |
|  | Reverse I | 5-CGG-TGG-TCA-CAT-CGT-GGG-T-3 |  |
|  | Reverse II | 5-GCT-GAA-GTA-CGT-VTC-GGT-GGC-3 |  |
|  | Reverse III | 5-GGT-GAA-GTA-GGT-GTC-CGT-GGC-3 |  |
|  | Probe | 5-FAM-TGG-TGC-AGT-TYG-CCC-G-MGB(NFQ)-3 |  |
| Human Coronavirus [7] | Forward Primer | 5’-GTTCTGATAAGGCACCATATAGG-3’ | NL63 |
|  | Reverse Primer | 5’-TTTAGGAGGCAAATCAACACG-3’ |  |
|  | Probe | 5’-TXR-CGCATACGCCAACGCTCTTGAACA-3’ |  |
|  | Forward Primer | 5’-CATACTCTGACGGTCACAATAATA-3’ | OC43 |
|  | Reverse Primer | 5’-ACCTTAGCAACAGTCATATAAGC-3’ |  |
|  | Probe | 5’-YAK-TGCCCAAGAATAGCCAGTACCTAGT-3’ |  |
|  | Forward Primer | 5’-TCCTACTAYTCAAGAAGCTATCC-3 | HKU1 |
|  | Reverse Primer | 5’-AATGAACGATTATTGGGTCCAC-3’ |  |
|  | Probe | 5’-CY5-TYCGCCTGGTACGATTTTGCCTCA-3’ |  |
|  | Forward Primer | 5’-CATACTATCAACCCATTCAACAAG-3’ | 229E |
|  | Reverse Primer | 5’-CACGGCAACTGTCATGTATT-3’ |  |
|  | Probe | 5’-FAM-ATGAACCTGAACACCTGAAGCCAATCTATG-3’ |  |
| Human Enterovirus [8] | Forward Primer | 5’-GGCCCCTGAATGCGGCTAATCC-3’ | 5’ NTR |
|  | Reverse Primer | 5’-GCGATTGTCACCATWAGCAGYCA-3’ |  |
|  | Probe | 5’-FAM-CCGACTACTTTGGGWGTCCGTGT-IBFQ-3’ |  |
| Respiratory Syncytial Virus A [9] | Forward Primer | 5’-AGATCAACTTCTGTCATCCAGCAA-3’ | Nucleocapsid |
|  | Reverse Primer | 5’-TTCTGCACATCATAATTAGGAGTATCAAT-3’ |  |
|  | Probe | 5’-FAM-CACCATCCAACGGAGCACAGGAGAT-TAMRA-3’ |  |
| Respiratory Syncytial Virus B [9] | Forward Primer | 5’-AAGATGCAAATCATAAATTCACAGGA-3’ | Nucleocapsid |
|  | Reverse Primer | 5’-TGATATCCAGCATCTTTAAGTATCTTTATAGTG-3’ |  |
|  | Probe | 5’-FAM-TTCCCTTCCTAACCTGGACATAGCATAT AACATACCT-TAMRA-3’ |  |
| Parainfluenza 1 [9] | Forward Primer | 5’-TGATTTAAACCCGGTAATTTCTCAT -3’ | Hemagglutinin-neuraminidase |
|  | Reverse Primer | 5’-CCTTGTTCCTGCAGCTATTACAGA-3’ |  |
|  | Probe | 5’-FAM-ACGACAACAGGAAATC-BHQ-3’ |  |
| Parainfluenza 2 [9] | Forward Primer | 5’-AGGACTATGAAAACCATTTACCTAAGTGA-3’ | Hemagglutinin-neuraminidase |
|  | Reverse Primer | 5’-AAGCAAGTCTCAGTTCAGCTAGATCA-3’ |  |
|  | Probe | 5’-FAM ATCAATCGCAAAAGCTGTTCAGTCACTGC TATAC-TAMRA-3’ |  |
| Parainfluenza 3 [9] | Forward Primer | 5’-TGATGAAAGATCAGATTATGCATATC -3 | Hemagglutinin-neuraminidase |
|  | Reverse Primer | 5’-CCGGGACACCCAGTTGTG -3’ |  |
|  | Probe | 5’-FAM-TGGACCAGGGATATACTACAAAGGCAAAAT AAT ATT TCT C-TAMRA-3’ |  |
| Parainfluenza 4 [9] | Forward Primer | 5’-CAAAYGATCCACAGCAAAGATTC -3’ | Nucleocapsid |
|  | Reverse Primer | 5’-ATGTGGCCTGTAAGGAAAGCA -3’ |  |
|  | Probe | 5’-FAM-GTATCATCATCTGCCAAATCGGCAAT TAAACA-TAMRA -3’ |  |

Supplementary Table 3: Risk Factors for Molecular Detection of Adenovirus (AdV)

| Risk Factor | Total N | AdV + (%) | Unadjusted OR  (95% CI) | Adjusted OR^†^  (95% CI) |
| --- | --- | --- | --- | --- |
| Approximate age quartiles |  |  |  |  |
| 1 month-1 year | 179^*^ | 34 (19.0) | 7.3 (2.8, 19.2) | 6.3 (2.3, 17.1) |
| 1-2 years | 105 | 16 (15.2) | 5.6 (2.0, 15.8) | 5.0 (1.7, 14.6) |
| 2-18 years | 154 | 7 (4.5) | 1.5 (0.5, 4.8) | 1.4 (0.4, 4.5) |
| >18 years | 161 | 5 (3.1) | Ref. | Ref. |
| Month |  |  |  |  |
| June 15-July 14 | 97 | 7 (7.2) | 3.5 (0.4, 29.3) | 2.8 (0.3, 23.9) |
| July 15-Aug 14 | 51 | 2 (3.9) | 1.8 (0.2, 21.0) | 2.1 (0.2, 24.2) |
| Aug 15-Sept 14 | 41 | 1 (2.4) | 1.1 (0.1, 18.6) | 1.1 (0.1, 19.2) |
| Sept 15-Oct 14 | 53 | 4 (7.5) | 3.7 (0.4, 34.1) | 3.1 (0.2, 29.4) |
| Nov 15-Dec 14 | 42 | 0 (0.0) | -- | -- |
| Dec 15-Jan 14 | 43 | 2 (4.7) | 2.2 (.2, 25.1) | 2.8 (0.2, 33.5) |
| Jan 15-Feb 14 | 71 | 10 (14.1) | 7.4 (0.9, 59.7) | 7.3 (0.9, 61.1) |
| Feb 15-Mar 14 | 40 | 5 (12.5) | 6.4 (0.7, 57.6) | 5.0 (0.5, 46.0) |
| Mar 15-Apr 14 | 43 | 8 (18.6) | 10.3 (1.2, 86.1) | 8.2 (0.9, 70.6) |
| Apr 15-May 14 | 72 | 22 (30.6) | 19.8 (2.6, 152.9) | 16.9 (2.1, 133.4) |
| Oct 15-Nov 14 | 46^*^ | 1 (2.2) | Ref. | Ref. |

^*^ One pediatric patient specimen destroyed, assay results out of n=599

**^†^**Adjusted for age quartile and month of enrolment

Supplementary Table 4: Risk Factors for Molecular Detection of Influenza A Virus (IAV)

| Risk Factor | Total N | IAV + (%) | Unadjusted OR^†^  (95% CI) |
| --- | --- | --- | --- |
| Month |  |  |  |
| June 15-July 14 | 97 | 14 (14.4) | 4.3 (0.9, 19.7) |
| July 15-Aug 14 | 51 | 5 (9.8) | 2.8 (0.5, 15.0) |
| Aug 15-Sept 14 | 41 | 3 (7.3) | 2.0 (0.3, 12.6) |
| Oct 15-Nov 14 | 46^*^ | 0 (0.0) | -- |
| Nov 15-Dec 14 | 42 | 0 (0.0) | -- |
| Dec 15-Jan 14 | 43 | 6 (14.0) | 4.1 (0.8, 21.6) |
| Jan 15-Feb 14 | 71 | 16 (22.5) | 7.4 (1.6, 33.9) |
| Feb 15-Mar 14 | 40 | 13 (32.5) | 12.3 (2.6, 58.4) |
| Mar 15-Apr 14 | 43 | 3 (7.0) | 1.9 (0.3, 12.0) |
| Apr 15-May 14 | 72 | 0 (0.0) | -- |
| Sept 15-Oct 14 | 53 | 2 (3.8) | Ref. |

**^*^** One pediatric patient specimen destroyed, assay results out of n=599

**^†^** There were no additional covariates to perform adjusted modeling

Supplementary Table 5: Risk Factors for Molecular Detection of Enterovirus (EV)

| Risk Factor | Total N | EV + (%) | Unadjusted OR^†^  (95% CI) |
| --- | --- | --- | --- |
| Hospital |  |  |  |
| Kapit | 211 | 14 (6.6) | 2.4 (1.1, 5.5) |
| Sibu | 388^*^ | 11 (2.8) | Ref. |

**^*^** One pediatric patient specimen destroyed, assay results out of n=599

**^†^** There were no additional covariates to perform adjusted modeling

Supplementary Table 6: Risk Factors for Molecular Detection of Respiratory Syncytial Virus A (RSV-A) Among Pediatric Patients

| Risk Factor | Total N | RSV-A + (%) | Unadjusted OR  (95% CI) | Adjusted OR^†^  (95% CI) |  |
| --- | --- | --- | --- | --- | --- |
| Approximate age quartiles |  |  |  |  |  |
| 0-0.67 years | 109^*^ | 26 (23.9) | 6.5 (2.4, 17.7) | 6.7 (2.4, 18.8) |  |
| 0.68-1.32 years | 108 | 25 (23.1) | 6.3 (2.3, 17.1) | 7.0 (2.5, 19.9) |  |
| 1.33-2.78 years | 109 | 26 (23.9) | 6.5 (2.4, 17.7) | 7.0 (2.5, 19.7) |  |
| 2.79-18 years | 109 | 5 (4.6) | Ref. | Ref. |  |
| Household size quartiles |  |  |  |  |  |
| 0-3 | 97 | 21 (21.6) | 1.6 (0.8, 3.2) | 2.0 (0.9, 4.5) |  |
| 4-5 | 132^*^ | 19 (14.4) | 1.0 (0.5, 2.0) | 1.1 (0.5, 2.5) |  |
| ≥ 8 | 102 | 27 (26.5) | 2.0 (1.0, 4.1) | 2.2 (1.1, 4.7) |  |
| 6-7 | 107 | 16 (15.0) | Ref. | Ref. |  |
| Month |  |  |  |  |  |
| June 15-July 14 | 75 | 27 (36.0) | 8.7 (1.9, 39.3) | 9.3 (2.0, 43.1) |  |
| July 15-Aug 14 | 37 | 9 (24.3) | 5.0 (1.0, 25.1) | 6.2 (1.2, 32.7) |  |
| Aug 15-Sept 14 | 25 | 5 (20.0) | 3.9 (0.7, 21.9) | 4.7 (0.8, 27.8) |  |
| Sept 15-Oct 14 | 43 | 8 (18.6) | 3.5 (0.7, 18.0) | 3.9 (0.7, 20.6) |  |
| Oct 15-Nov 14 | 26 | 7 (26.9) | 6.0 (1.1, 32.2) | 7.1 (1.2, 40.0) |  |
| Nov 15-Dec 14 | 27 | 2 (7.4) | 1.2 (0.2, 9.4) | 1.4 (0.2, 11.0) |  |
| Dec 15-Jan 14 | 30 | 2 (6.7) | 1.1 (0.1, 8.4) | 1.8 (0.2, 14.3) |  |
| Jan 15-Feb 14 | 49 | 5 (10.2) | 1.8 (0.3, 9.7) | 1.8 (0.2, 10.0) |  |
| Mar 15-Apr 14 | 38 | 9 (23.7) | 4.8 (1.0, 24.2) | 4.5 (0.9, 23.5) |  |
| Apr 15-May 14 | 56 | 7 (12.5) | 2.2 (0.4, 11.4) | 2.2 (0.4, 11.3) |  |
| Feb 15-Mar 14 | 33 | 2 (06.1) | Ref. | Ref. |  |

^*^ One pediatric patient specimen destroyed, assay results out of n=438

**^†^** Adjusted for pediatric age quartiles, pediatric household size quartile, pediatric month of enrollment

Supplementary Table 7: Risk Factors for Molecular Detection of Influenza B Virus (IBV)

| Risk Factor | Total N | IBV + (%) | Unadjusted OR^†^  (95% CI) |
| --- | --- | --- | --- |
| Exposure to Cat |  |  |  |
| Contact | 141^*^ | 8 (5.7) | 5.4 (1.8-16.9) |
| No Contact | 458 | 5 (1.1) | Ref. |

**^*^** One pediatric patient specimen destroyed, assay results out of n=599

**^†^** There were no additional covariates to perform adjusted modeling

**Figure Legends**

Supplementary Figure 1. Map of Sibu and Kapit Hospital sites in Sarawak, Malaysia on the island of Borneo. The towns of Sibu and Kapit are located approximately two degrees north of the equator. Created using Google Maps.

Supplementary Figure 2. Average Temperature and Humidity in Kuching, Sarawak, Malaysia, June 2017-May 2018. Source: Kuching Historical Weather, WorldWeatherOnline.com [10].

Supplementary Figure 3. Respiratory Syncytial Virus type A and B detected at Sibu and Kapit Hospitals between June 2017 and May 2018. The prevalence of RSV-A and RSV-B throughout the year suggests there is seasonal variation in the drier spring months.


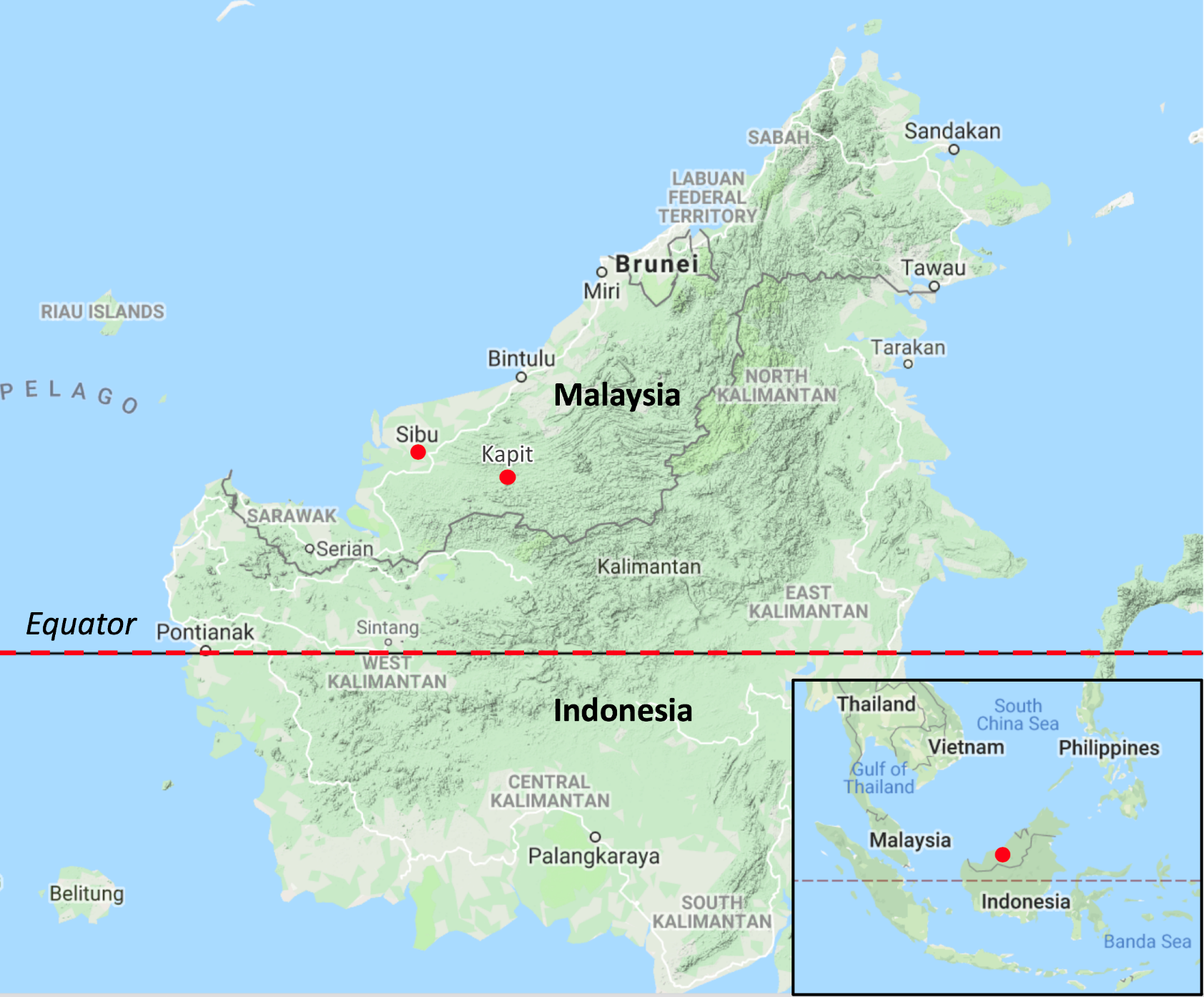


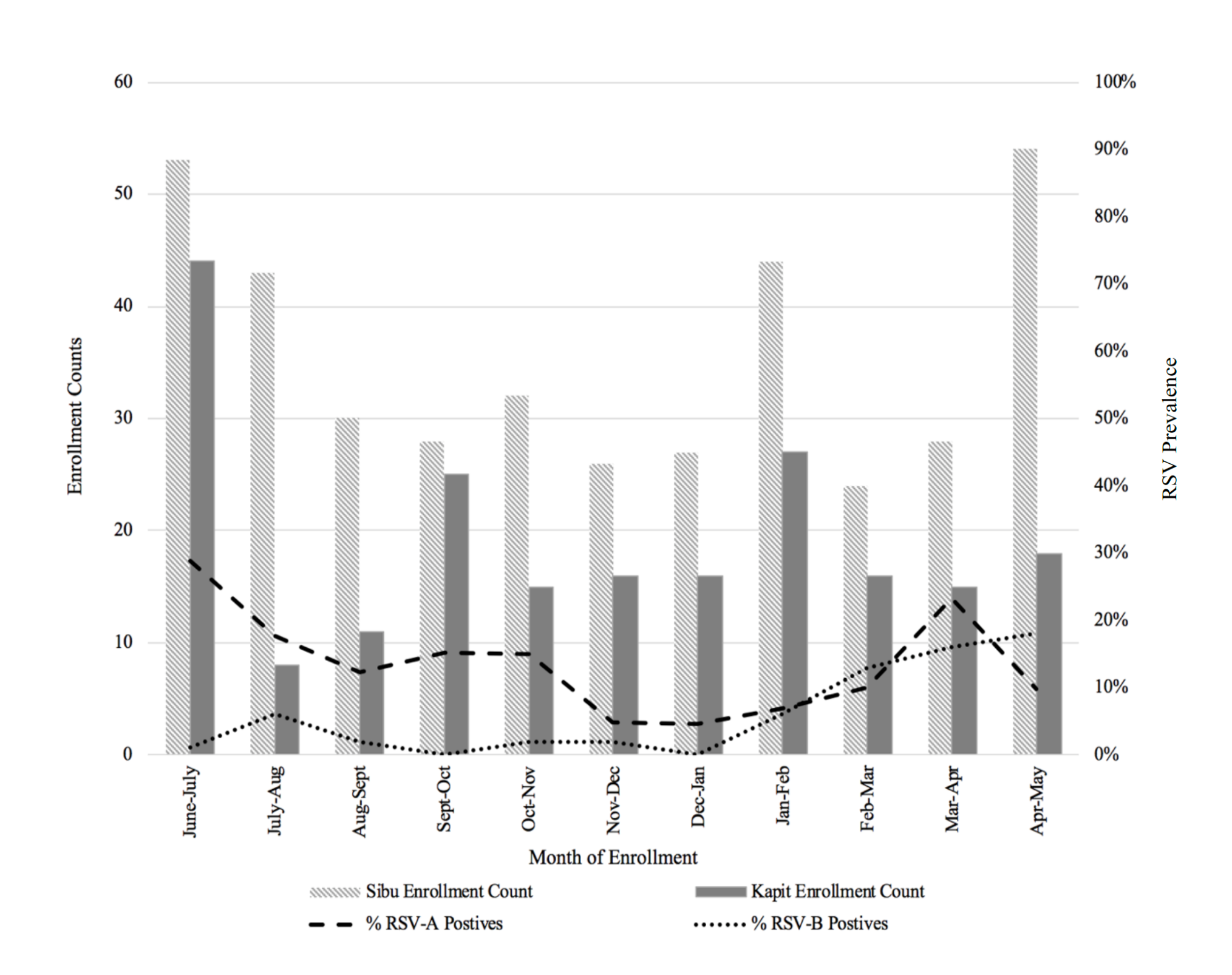


**REFERENCES**

1. Jain, S., et al., *Community-Acquired Pneumonia Requiring Hospitalization among U.S. Children.* New England Journal of Medicine, 2015. **372**(9): p. 835-845.

2. Jain, S., et al., *Community-Acquired Pneumonia Requiring Hospitalization among U.S. Adults.* New England Journal of Medicine, 2015. **373**(5): p. 415-427.

3. *WHO | WHO information for molecular diagnosis of influenza virus - update.* WHO, 2017.

4. Pabbaraju, K., et al., *Detection of influenza C virus by a real‐time RT‐PCR assay.* Influenza and Other Respiratory Viruses, 2013. **7**(6): p. 954-960.

5. Hause, B.M., et al., *Isolation of a Novel Swine Influenza Virus from Oklahoma in 2011 Which Is Distantly Related to Human Influenza C Viruses.* PLoS Pathogens, 2013. **9**(2): p. e1003176.

6. Bil-Lula, I., et al., *Improved real-time PCR assay for detection and quantification of all 54 known types of human adenoviruses in clinical samples.* Medical Science Monitor : International Medical Journal of Experimental and Clinical Research, 2012. **18**(6): p. BR221-BR228.

7. Loens, K., et al., *Performance of Different Mono- and Multiplex Nucleic Acid Amplification Tests on a Multipathogen External Quality Assessment Panel.* Journal of Clinical Microbiology, 2012. **50**(3): p. 977-987.

8. Oberste, M.S., et al., *Characterizing the Picornavirus Landscape among Synanthropic Nonhuman Primates in Bangladesh, 2007 to 2008.* Journal of Virology, 2013. **87**(1): p. 558-571.

9. van de Pol, A.C., et al., *Increased Detection of Respiratory Syncytial Virus, Influenza Viruses, Parainfluenza Viruses, and Adenoviruses with Real-Time PCR in Samples from Patients with Respiratory Symptoms.* Journal of Clinical Microbiology, 2007. **45**(7): p. 2260-2262.

10. *Kuching Historical Weather*. 2018 [cited 2018 November 29]; Available from: <https://www.worldweatheronline.com/lang/en-nz/kuching-weather/sarawak/my.aspx>.
