# Supplementary figures and images for: High Prevalence of Viral Infections Among Hospitalized Pneumonia Patients in Equatorial Sarawak, Malaysia
Source: Open Forum Infect Dis. 2019 Feb 13;6(3):ofz074. doi: 10.1093/ofid/ofz074 (PMC6440682; doi:10.1093/ofid/ofz074)

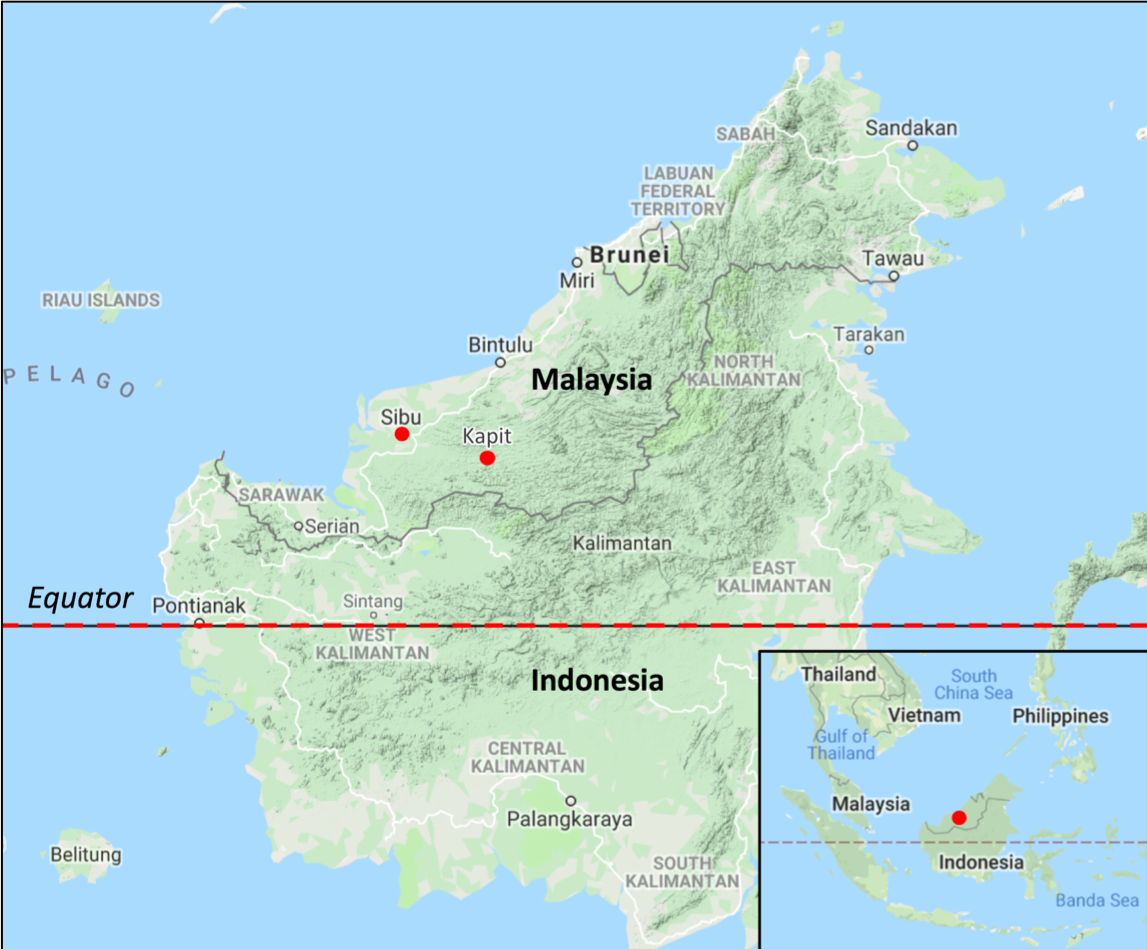

Supplement: ofz074_suppl_supplementary_figure_1 [file ofz074_suppl_supplementary_figure_1.png]

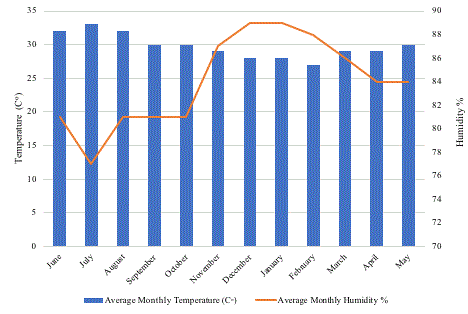

Supplement: ofz074_suppl_supplementary_figure_2 [file ofz074_suppl_supplementary_figure_2.png]

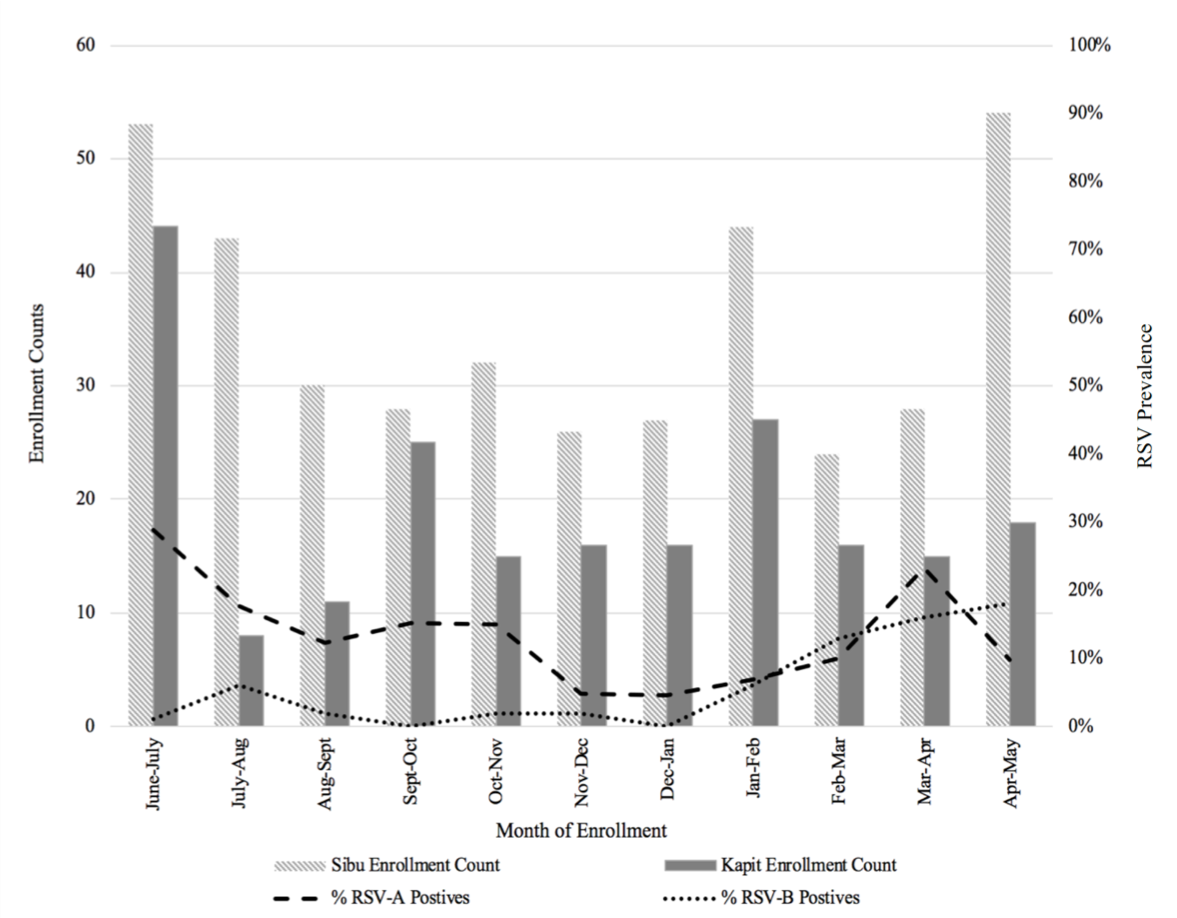

Supplement: ofz074_suppl_supplementary_figure_3 [file ofz074_suppl_supplementary_figure_3.png]
