# Supplementary material for: High Prevalence of Viral Infections Among Hospitalized Pneumonia Patients in Equatorial Sarawak, Malaysia
Source: Open Forum Infect Dis. 2019 Feb 13;6(3):ofz074. doi: 10.1093/ofid/ofz074 (PMC6440682; doi:10.1093/ofid/ofz074)
Supplement: ofz074_suppl_supplementary_table_1 [file ofz074_suppl_supplementary_table_1.docx]

Supplementary Table 1: Inclusion and exclusion criteria checklist used by medical officers while assessing patients for enrollment eligibility

| Inclusion Criteria Children (1 month to 18 years) | Inclusion Criteria Adults (18 years or more) |
| --- | --- |
| - They were admitted to Sibu or Kapit hospital; - Have evidence of acute infection, defined as reported fever or chills, documented fever or hypothermia, or leukocytosis or leukopenia; - Have evidence of an acute respiratory illness, defined as new cough or sputum production, chest pain, dyspnea, tachypnea, abnormal lung examination, or respiratory failure; - A parent or legal guardian provides written informed consent. In addition to parental consent, signed assent document will be sought from children 7 to 18 years of age. - The evidence of illness is consistent with pneumonia as assessed by means of chest radiography within 72 hours before or after admission. | - They were admitted to Sibu or Kapit hospital on the basis of a clinical assessment by the treating clinician; - Have evidence of acute infection, defined as reported fever or chills, documented fever or hypothermia, leukocytosis or leukopenia, or new altered mental status; - Have evidence of an acute respiratory illness, defined as new cough or sputum production, chest pain, dyspnea, tachypnea, abnormal lung examination, or respiratory failure; - Have evidence consistent with pneumonia as assessed by means of chest radiography by the clinical team within 48 hours before or after admission. |
| Exclusion Criteria Children (1 month to 18 years) | Exclusion Criteria Adults (18 years or more) |
| - If they had been hospitalized recently (<7 days for immunocompetent children and <90 days for immunosuppressed children) - If they had already been enrolled in this study within the previous 28 days - If they resided in an extended-care facility - If they had an alternative diagnosis of a respiratory disorder - If they are newborns who never left the hospital - If they have a tracheostomy tube - If they have cystic fibrosis or - If they have cancer with neutropenia - If they have received a solid-organ or hematopoietic stem-cell transplant within the previous 90 days - If they have active graft-versus-host disease or bronchiolitis obliterans - If they have human immunodeficiency virus infection with a CD4 cell count of less than 200 per cubic millimeter (or a percentage of CD4 cells <14%). | - If they had been hospitalized recently (<28 days for immunocompetent patients and <90 days for immunosuppressed patients), - If they have already been enrolled in this study within the previous 28 days - If they were functionally dependent nursing home residents, - If they have a clear alternative diagnosis - If they have undergone tracheotomy - If they have a percutaneous endoscopic gastrostomy tube - If they have cystic fibrosis - If they have cancer with neutropenia, - If they have received a solid-organ or hematopoietic stem-cell transplant within the previous 90 days, - If they have active graft-versus-host disease - If they have bronchiolitis obliterans - If they have human immunodeficiency virus infection with a CD4 cell count of less than 200 per cubic millimeter. |

Adapted from: Jain S et al. 2015;372(9) [1] and Jain S et al. 2015;373(5) [2].
