# Supplementary material for: High Prevalence of Viral Infections Among Hospitalized Pneumonia Patients in Equatorial Sarawak, Malaysia
Source: Open Forum Infect Dis. 2019 Feb 13;6(3):ofz074. doi: 10.1093/ofid/ofz074 (PMC6440682; doi:10.1093/ofid/ofz074)
Supplement: ofz074_suppl_supplementary_table_2 [file ofz074_suppl_supplementary_table_2.docx]

Supplementary Table 2: Primers and probes sequences and gene target region for all rRT-PCR and rPRC assays. All assays were adapted from the cited references at Duke University.

| ***Virus Assay*** | ***Function*** | ***Sequence*** | ***Target Gene*** |
| --- | --- | --- | --- |
| Influenza A [3] | Forward Primer | 5-GAC-CRA-TCC-TGT-CAC-CTC-TGA-C-3 | Matrix |
|  | Reverse Primer | 5-AGG-GCA-TTY-TGG-ACA-AAK-CGT-CTA-3 |  |
|  | Probe | 5-FAM-TGC-AGT-CCT-CGC-TCA-CTG-GGC-ACG-BHQ 1-3 |  |
| Influenza B [3] | Forward Primer | 5-TCC-TCA-AYT-CAC-TCT-TCG-AGC-G-3 | Matrix |
|  | Reverse Primer | 5-CGG-TGC-TCT-TGA-CCA-AAT-TGG-3 |  |
|  | Probe | 5-FAM-CCA-ATT-CGA-GCA-GCT-GAA-ACT-GCG-GTG-BHQ 1-3 |  |
| Influenza C [4] | Forward Primer | 5’-TGGGAGAGATGGTGTGGAGATA-3’ | Matrix |
|  | Reverse Primer | 5’-TCTTTTTCCATCGAGTCAATTTCA-3’ |  |
|  | Probe | 5’-FAM-AAAGACCACAATTATGC-IBFQ-3’ |  |
| Influenza D [5] | Forward Primer | 5’-GCTGTTTGCAAGTTGATGGG-3’ | PB1 |
|  | Reverse Primer | 5’-TGAAAGCAGGTAACTCCAAGG-3’ |  |
|  | Probe | 5’-FAM-TTCAGGCAAGCACCCGTAGGATT-IBFQ-3’ |  |
| Human Adenovirus [6] | Forward | 5-CAG-GAC-GCY-TCG-GAG-TAC-CTG-A-3 | Hexon |
|  | Reverse I | 5-CGG-TGG-TCA-CAT-CGT-GGG-T-3 |  |
|  | Reverse II | 5-GCT-GAA-GTA-CGT-VTC-GGT-GGC-3 |  |
|  | Reverse III | 5-GGT-GAA-GTA-GGT-GTC-CGT-GGC-3 |  |
|  | Probe | 5-FAM-TGG-TGC-AGT-TYG-CCC-G-MGB(NFQ)-3 |  |
| Human Coronavirus [7] | Forward Primer | 5’-GTTCTGATAAGGCACCATATAGG-3’ | NL63 |
|  | Reverse Primer | 5’-TTTAGGAGGCAAATCAACACG-3’ |  |
|  | Probe | 5’-TXR-CGCATACGCCAACGCTCTTGAACA-3’ |  |
|  | Forward Primer | 5’-CATACTCTGACGGTCACAATAATA-3’ | OC43 |
|  | Reverse Primer | 5’-ACCTTAGCAACAGTCATATAAGC-3’ |  |
|  | Probe | 5’-YAK-TGCCCAAGAATAGCCAGTACCTAGT-3’ |  |
|  | Forward Primer | 5’-TCCTACTAYTCAAGAAGCTATCC-3 | HKU1 |
|  | Reverse Primer | 5’-AATGAACGATTATTGGGTCCAC-3’ |  |
|  | Probe | 5’-CY5-TYCGCCTGGTACGATTTTGCCTCA-3’ |  |
|  | Forward Primer | 5’-CATACTATCAACCCATTCAACAAG-3’ | 229E |
|  | Reverse Primer | 5’-CACGGCAACTGTCATGTATT-3’ |  |
|  | Probe | 5’-FAM-ATGAACCTGAACACCTGAAGCCAATCTATG-3’ |  |
| Human Enterovirus [8] | Forward Primer | 5’-GGCCCCTGAATGCGGCTAATCC-3’ | 5’ NTR |
|  | Reverse Primer | 5’-GCGATTGTCACCATWAGCAGYCA-3’ |  |
|  | Probe | 5’-FAM-CCGACTACTTTGGGWGTCCGTGT-IBFQ-3’ |  |
| Respiratory Syncytial Virus A [9] | Forward Primer | 5’-AGATCAACTTCTGTCATCCAGCAA-3’ | Nucleocapsid |
|  | Reverse Primer | 5’-TTCTGCACATCATAATTAGGAGTATCAAT-3’ |  |
|  | Probe | 5’-FAM-CACCATCCAACGGAGCACAGGAGAT-TAMRA-3’ |  |
| Respiratory Syncytial Virus B [9] | Forward Primer | 5’-AAGATGCAAATCATAAATTCACAGGA-3’ | Nucleocapsid |
|  | Reverse Primer | 5’-TGATATCCAGCATCTTTAAGTATCTTTATAGTG-3’ |  |
|  | Probe | 5’-FAM-TTCCCTTCCTAACCTGGACATAGCATAT AACATACCT-TAMRA-3’ |  |
| Parainfluenza 1 [9] | Forward Primer | 5’-TGATTTAAACCCGGTAATTTCTCAT -3’ | Hemagglutinin-neuraminidase |
|  | Reverse Primer | 5’-CCTTGTTCCTGCAGCTATTACAGA-3’ |  |
|  | Probe | 5’-FAM-ACGACAACAGGAAATC-BHQ-3’ |  |
| Parainfluenza 2 [9] | Forward Primer | 5’-AGGACTATGAAAACCATTTACCTAAGTGA-3’ | Hemagglutinin-neuraminidase |
|  | Reverse Primer | 5’-AAGCAAGTCTCAGTTCAGCTAGATCA-3’ |  |
|  | Probe | 5’-FAM ATCAATCGCAAAAGCTGTTCAGTCACTGC TATAC-TAMRA-3’ |  |
| Parainfluenza 3 [9] | Forward Primer | 5’-TGATGAAAGATCAGATTATGCATATC -3 | Hemagglutinin-neuraminidase |
|  | Reverse Primer | 5’-CCGGGACACCCAGTTGTG -3’ |  |
|  | Probe | 5’-FAM-TGGACCAGGGATATACTACAAAGGCAAAAT AAT ATT TCT C-TAMRA-3’ |  |
| Parainfluenza 4 [9] | Forward Primer | 5’-CAAAYGATCCACAGCAAAGATTC -3’ | Nucleocapsid |
|  | Reverse Primer | 5’-ATGTGGCCTGTAAGGAAAGCA -3’ |  |
|  | Probe | 5’-FAM-GTATCATCATCTGCCAAATCGGCAAT TAAACA-TAMRA -3’ |  |
