# Supplementary material for: High Prevalence of Viral Infections Among Hospitalized Pneumonia Patients in Equatorial Sarawak, Malaysia
Source: Open Forum Infect Dis. 2019 Feb 13;6(3):ofz074. doi: 10.1093/ofid/ofz074 (PMC6440682; doi:10.1093/ofid/ofz074)
Supplement: ofz074_suppl_supplementary_table_3 [file ofz074_suppl_supplementary_table_3.docx]

Supplementary Table 3: Risk Factors for Molecular Detection of Adenovirus (AdV)

| Risk Factor | Total N | AdV + (%) | Unadjusted OR  (95% CI) | Adjusted OR^†^  (95% CI) |
| --- | --- | --- | --- | --- |
| Approximate age quartiles |  |  |  |  |
| 1 month-1 year | 179^*^ | 34 (19.0) | 7.3 (2.8, 19.2) | 6.3 (2.3, 17.1) |
| 1-2 years | 105 | 16 (15.2) | 5.6 (2.0, 15.8) | 5.0 (1.7, 14.6) |
| 2-18 years | 154 | 7 (4.5) | 1.5 (0.5, 4.8) | 1.4 (0.4, 4.5) |
| >18 years | 161 | 5 (3.1) | Ref. | Ref. |
| Month |  |  |  |  |
| June 15-July 14 | 97 | 7 (7.2) | 3.5 (0.4, 29.3) | 2.8 (0.3, 23.9) |
| July 15-Aug 14 | 51 | 2 (3.9) | 1.8 (0.2, 21.0) | 2.1 (0.2, 24.2) |
| Aug 15-Sept 14 | 41 | 1 (2.4) | 1.1 (0.1, 18.6) | 1.1 (0.1, 19.2) |
| Sept 15-Oct 14 | 53 | 4 (7.5) | 3.7 (0.4, 34.1) | 3.1 (0.2, 29.4) |
| Nov 15-Dec 14 | 42 | 0 (0.0) | -- | -- |
| Dec 15-Jan 14 | 43 | 2 (4.7) | 2.2 (.2, 25.1) | 2.8 (0.2, 33.5) |
| Jan 15-Feb 14 | 71 | 10 (14.1) | 7.4 (0.9, 59.7) | 7.3 (0.9, 61.1) |
| Feb 15-Mar 14 | 40 | 5 (12.5) | 6.4 (0.7, 57.6) | 5.0 (0.5, 46.0) |
| Mar 15-Apr 14 | 43 | 8 (18.6) | 10.3 (1.2, 86.1) | 8.2 (0.9, 70.6) |
| Apr 15-May 14 | 72 | 22 (30.6) | 19.8 (2.6, 152.9) | 16.9 (2.1, 133.4) |
| Oct 15-Nov 14 | 46^*^ | 1 (2.2) | Ref. | Ref. |

^*^ One pediatric patient specimen destroyed, assay results out of n=599

**^†^**Adjusted for age quartile and month of enrolment
