# Supplementary material for: High Prevalence of Viral Infections Among Hospitalized Pneumonia Patients in Equatorial Sarawak, Malaysia
Source: Open Forum Infect Dis. 2019 Feb 13;6(3):ofz074. doi: 10.1093/ofid/ofz074 (PMC6440682; doi:10.1093/ofid/ofz074)
Supplement: ofz074_suppl_supplementary_table_4 [file ofz074_suppl_supplementary_table_4.docx]

Supplementary Table 4: Risk Factors for Molecular Detection of Influenza A Virus (IAV)

| Risk Factor | Total N | IAV + (%) | Unadjusted OR^†^  (95% CI) |
| --- | --- | --- | --- |
| Month |  |  |  |
| June 15-July 14 | 97 | 14 (14.4) | 4.3 (0.9, 19.7) |
| July 15-Aug 14 | 51 | 5 (9.8) | 2.8 (0.5, 15.0) |
| Aug 15-Sept 14 | 41 | 3 (7.3) | 2.0 (0.3, 12.6) |
| Oct 15-Nov 14 | 46^*^ | 0 (0.0) | -- |
| Nov 15-Dec 14 | 42 | 0 (0.0) | -- |
| Dec 15-Jan 14 | 43 | 6 (14.0) | 4.1 (0.8, 21.6) |
| Jan 15-Feb 14 | 71 | 16 (22.5) | 7.4 (1.6, 33.9) |
| Feb 15-Mar 14 | 40 | 13 (32.5) | 12.3 (2.6, 58.4) |
| Mar 15-Apr 14 | 43 | 3 (7.0) | 1.9 (0.3, 12.0) |
| Apr 15-May 14 | 72 | 0 (0.0) | -- |
| Sept 15-Oct 14 | 53 | 2 (3.8) | Ref. |

**^*^** One pediatric patient specimen destroyed, assay results out of n=599

**^†^** There were no additional covariates to perform adjusted modeling
