# Supplementary material for: High Prevalence of Viral Infections Among Hospitalized Pneumonia Patients in Equatorial Sarawak, Malaysia
Source: Open Forum Infect Dis. 2019 Feb 13;6(3):ofz074. doi: 10.1093/ofid/ofz074 (PMC6440682; doi:10.1093/ofid/ofz074)
Supplement: ofz074_suppl_supplementary_table_5 [file ofz074_suppl_supplementary_table_5.docx]

Supplementary Table 5: Risk Factors for Molecular Detection of Enterovirus (EV)

| Risk Factor | Total N | EV + (%) | Unadjusted OR^†^  (95% CI) |
| --- | --- | --- | --- |
| Hospital |  |  |  |
| Kapit | 211 | 14 (6.6) | 2.4 (1.1, 5.5) |
| Sibu | 388^*^ | 11 (2.8) | Ref. |

**^*^** One pediatric patient specimen destroyed, assay results out of n=599

**^†^** There were no additional covariates to perform adjusted modeling
