# Supplementary material for: High Prevalence of Viral Infections Among Hospitalized Pneumonia Patients in Equatorial Sarawak, Malaysia
Source: Open Forum Infect Dis. 2019 Feb 13;6(3):ofz074. doi: 10.1093/ofid/ofz074 (PMC6440682; doi:10.1093/ofid/ofz074)
Supplement: ofz074_suppl_supplementary_table_6 [file ofz074_suppl_supplementary_table_6.docx]

Supplementary Table 6: Risk Factors for Molecular Detection of Respiratory Syncytial Virus A (RSV-A) Among Pediatric Patients

| Risk Factor | Total N | RSV-A + (%) | Unadjusted OR  (95% CI) | Adjusted OR^†^  (95% CI) |  |
| --- | --- | --- | --- | --- | --- |
| Approximate age quartiles |  |  |  |  |  |
| 0-0.67 years | 109^*^ | 26 (23.9) | 6.5 (2.4, 17.7) | 6.7 (2.4, 18.8) |  |
| 0.68-1.32 years | 108 | 25 (23.1) | 6.3 (2.3, 17.1) | 7.0 (2.5, 19.9) |  |
| 1.33-2.78 years | 109 | 26 (23.9) | 6.5 (2.4, 17.7) | 7.0 (2.5, 19.7) |  |
| 2.79-18 years | 109 | 5 (4.6) | Ref. | Ref. |  |
| Household size quartiles |  |  |  |  |  |
| 0-3 | 97 | 21 (21.6) | 1.6 (0.8, 3.2) | 2.0 (0.9, 4.5) |  |
| 4-5 | 132^*^ | 19 (14.4) | 1.0 (0.5, 2.0) | 1.1 (0.5, 2.5) |  |
| ≥ 8 | 102 | 27 (26.5) | 2.0 (1.0, 4.1) | 2.2 (1.1, 4.7) |  |
| 6-7 | 107 | 16 (15.0) | Ref. | Ref. |  |
| Month |  |  |  |  |  |
| June 15-July 14 | 75 | 27 (36.0) | 8.7 (1.9, 39.3) | 9.3 (2.0, 43.1) |  |
| July 15-Aug 14 | 37 | 9 (24.3) | 5.0 (1.0, 25.1) | 6.2 (1.2, 32.7) |  |
| Aug 15-Sept 14 | 25 | 5 (20.0) | 3.9 (0.7, 21.9) | 4.7 (0.8, 27.8) |  |
| Sept 15-Oct 14 | 43 | 8 (18.6) | 3.5 (0.7, 18.0) | 3.9 (0.7, 20.6) |  |
| Oct 15-Nov 14 | 26 | 7 (26.9) | 6.0 (1.1, 32.2) | 7.1 (1.2, 40.0) |  |
| Nov 15-Dec 14 | 27 | 2 (7.4) | 1.2 (0.2, 9.4) | 1.4 (0.2, 11.0) |  |
| Dec 15-Jan 14 | 30 | 2 (6.7) | 1.1 (0.1, 8.4) | 1.8 (0.2, 14.3) |  |
| Jan 15-Feb 14 | 49 | 5 (10.2) | 1.8 (0.3, 9.7) | 1.8 (0.2, 10.0) |  |
| Mar 15-Apr 14 | 38 | 9 (23.7) | 4.8 (1.0, 24.2) | 4.5 (0.9, 23.5) |  |
| Apr 15-May 14 | 56 | 7 (12.5) | 2.2 (0.4, 11.4) | 2.2 (0.4, 11.3) |  |
| Feb 15-Mar 14 | 33 | 2 (06.1) | Ref. | Ref. |  |

^*^ One pediatric patient specimen destroyed, assay results out of n=438

**^†^** Adjusted for pediatric age quartiles, pediatric household size quartile, pediatric month of enrollment
