# Supplementary material for: High Prevalence of Viral Infections Among Hospitalized Pneumonia Patients in Equatorial Sarawak, Malaysia
Source: Open Forum Infect Dis. 2019 Feb 13;6(3):ofz074. doi: 10.1093/ofid/ofz074 (PMC6440682; doi:10.1093/ofid/ofz074)
Supplement: ofz074_suppl_supplementary_table_7 [file ofz074_suppl_supplementary_table_7.docx]

Supplementary Table 7: Risk Factors for Molecular Detection of Influenza B Virus (IBV)

| Risk Factor | Total N | IBV + (%) | Unadjusted OR^†^  (95% CI) |
| --- | --- | --- | --- |
| Exposure to Cat |  |  |  |
| Contact | 141^*^ | 8 (5.7) | 5.4 (1.8-16.9) |
| No Contact | 458 | 5 (1.1) | Ref. |

**^*^** One pediatric patient specimen destroyed, assay results out of n=599

**^†^** There were no additional covariates to perform adjusted modeling
